# Supplementary material for: Synergistically Enhanced Electrochemical Sensing of Food Adulterant in Milk Sample at Erbium Vanadate/Graphitic Carbon Nitride Composite
Source: Sensors (Basel). 2024 Mar 11;24(6):1808. doi: 10.3390/s24061808 (PMC10975317; doi:10.3390/s24061808)
Supplement: Supplementary file 1 [file sensors-24-01808-s001.zip › sensors-2846785-supplementary.pdf]

1.Experimental section

1.1. Chemicals and reagents

Erbium (III) nitrate pentahydrate ($ErH_{10}N_3O_{14}$), ammonium metavanadate (NH_4VO_3≥99% purity), and urea (CH_4N_2O) were purchased from Sigma Aldrich. Potassium chloride (KCl), potassium ferricyanide ($K_3Fe(CN)_6$), and potassium ferrocyanide ($K_4Fe(CN)_6.3H_2O$) were from Showa Chemical CO.LTD, Japan. Sodium phosphate dibasic and sodium dihydrogen phosphate (Na_2HPO_4 and NaH_2PO_4) were utilized to prepare 0.1 M (pH 7) PBS (phosphate buffer solution). All the electrochemical experiments were carried out using 0.1 M PBS (pH 7) as the supporting electrolyte.

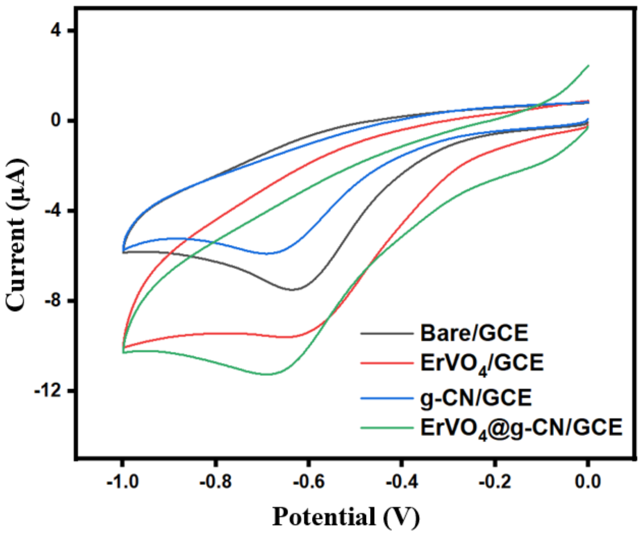

Figure S1. CV measurements of bare GCE, $ErVO_4$/GCE, g-CN/GCE, and $ErVO_4$@g-CN/GCE of composite in PBS (pH=7).

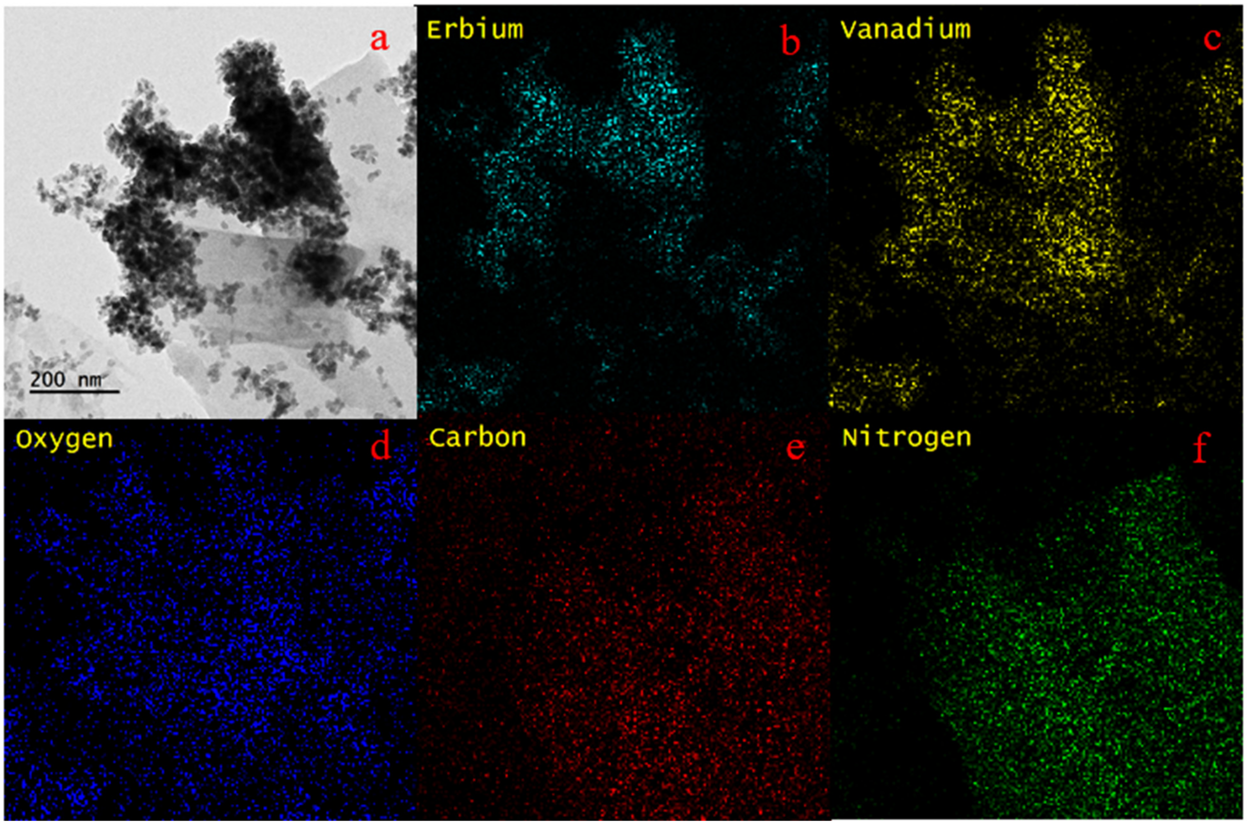

Figure S2. (a) HR-TEM image of $ErVO_4$@g-CN composite. **(b-f)** Elemental mapping of Er, V, O, C, and N.

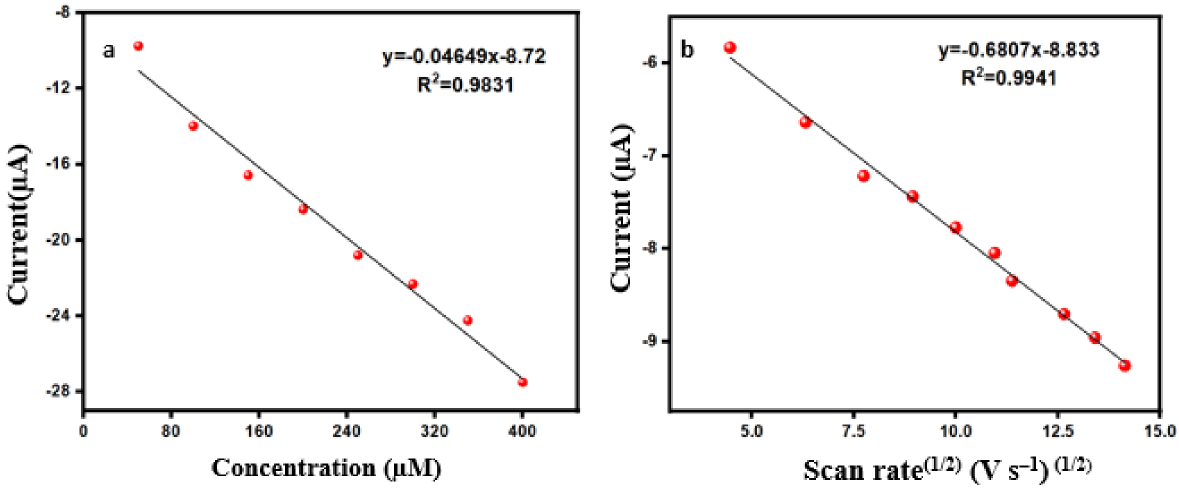

Figure S3. (a) Corresponding linear plot (for Figure (4g)) of current vs concentration. **(b)** corresponding calibration plot for (Figure (4h)) of current vs. different (scan rate)$^{1/2}$.

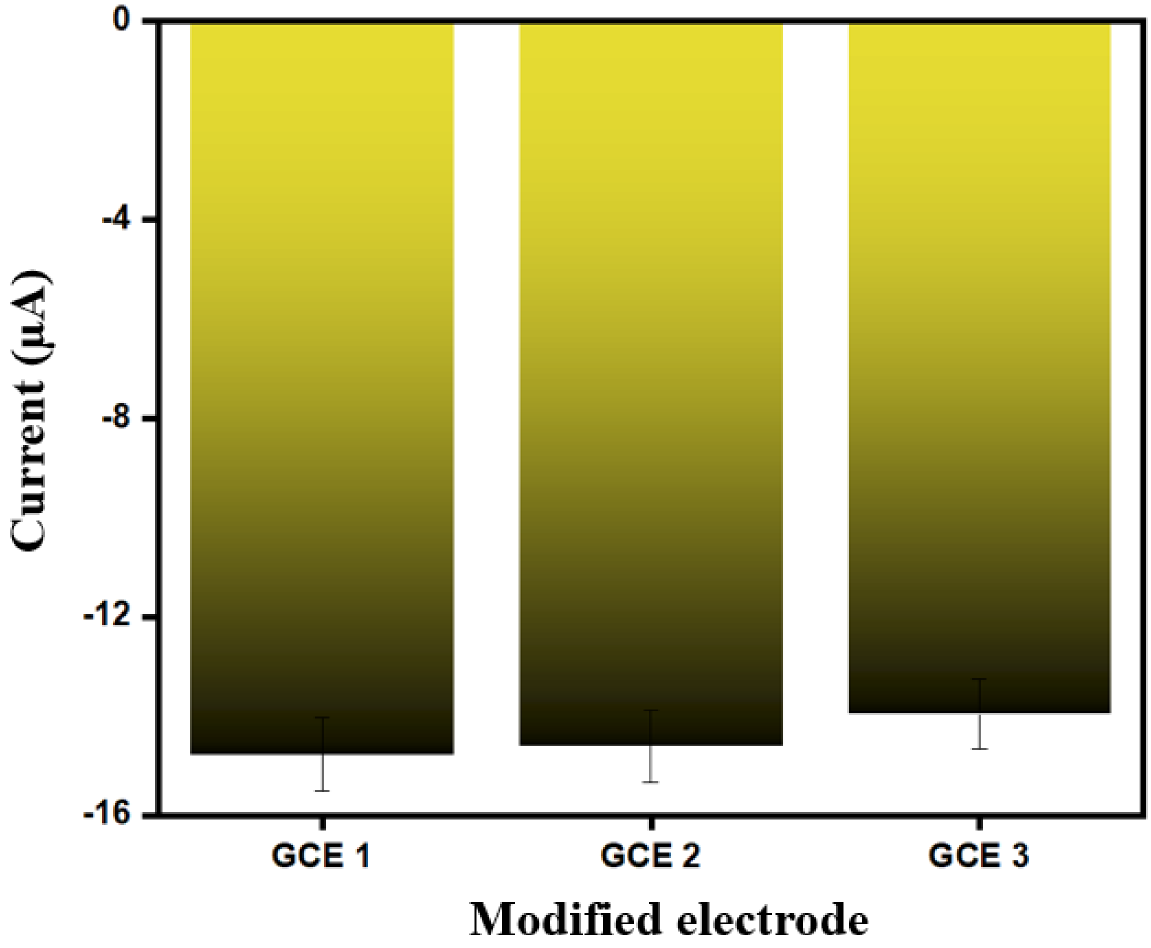

Figure S4. Corresponding bar graph for reproducibility in the presence of DMZ 100 μL and pH = 7 PBS.
